# Epigenetic Role of Histone Lysine Methyltransferase and Demethylase on the Expression of Transcription Factors Associated with the Epithelial-to-Mesenchymal Transition of Lung Adenocarcinoma Metastasis to the Brain

**DOI:** 10.3390/cancers12123632

**Published:** 2020-12-04

**Authors:** Young Min Lee, Seok Hyun Kim, Minseok S. Kim, Dae Cheol Kim, Eun Hee Lee, Ju Suk Lee, Sung-Hun Lee, Young Zoon Kim

**Affiliations:** 1 Division of Neuro Oncology, Department of Neurosurgery, Samsung Changwon Hospital, Sungkyunkwan University School of Medicine, Changwon 51353, Korea; mulsae@hanmail.net; 2 Division of Hematology and Medical Oncology, Department of Internal Medicine, Samsung Changwon Hospital, Sungkyunkwan University School of Medicine, Changwon 51353, Korea; tjrgus1@hanmail.net; 3 Department of New Biology, Well Aging Research Center, College of Transdisciplinary Studies, and Translational Responsive Medicine Center, Daegu Gyeongbuk Institute of Science and Technology, Daegu 42988, Korea; kms@dgist.ac.kr; 4 Department of Pathology, Dong-A University Hospital, Dong-A University College of Medicine, Busan 49201, Korea; freehand@donga.ac.kr; 5 Department of Pathology, Samsung Changwon Hospital, Sungkyunkwan University School of Medicine, Changwon 51353, Korea; dalgaebe@naver.com; 6 Department of Pediatrics, Samsung Changwon Hospital, Sungkyunkwan University School of Medicine, Changwon 51353, Korea; ljs8952194@nate.com; 7 Cancer Research Institute, Clinomics Inc., Suwon 16229, Korea; sunghun.lee76@gmail.com

**Keywords:** lung cancer, brain metastasis, epigenome, histone modification, epithelial-to-mesenchymal transition

## Abstract

**Simple Summary:**

The epithelial-to-mesenchymal transition (EMT) is an essential step for cancer metastasis to the brain. The transcription factors (TFs), which are associated with EMT, plays a major role during EMT. The objectives of this study were to investigate the epigenetic modification of EMT by regulating the expression of EMT-TFs during the metastasis of lung cancer into the brain. Several EMT-TFs such as Slug, Twist, ZEB1, and FOXC2 had higher immunoreactivity in brain metastasis than lung cancer. MLL4 (H3K4 methyltransferase) induces the expression of Slug, UTX (H3K36me3 demethylase) induces the expression of ZEB1, and EZH2 (H3K27 methyltransferase) suppressed the expression of Twist in the analysis of immunohistochemical staining and qRT-PCR for the 46 paired samples of lung cancer and its brain metastasis in individual patients. These results were also statistically significantly associated with the survival of the patients.

**Abstract:**

Purpose: The objective of this study was to investigate the epigenetic role of histone lysine methylation/demethylation on the expression of epithelial-to-mesenchymal transition (EMT) associated transcriptional factors (TFs) during the metastasis of lung adenocarcinoma to the brain. Methods: Paired samples of lung adenocarcinoma and brain metastasis (BM) were analyzed in 46 individual patients. Both samples were obtained by surgical resection or biopsy of the lung and brain. The paraffin-fixed formalin-embedded samples were obtained from the pathology archives in our institute. In samples of lung adenocarcinoma and BM, immunohistochemical staining was performed for epithelial markers, mesenchymal markers, EMT-TFs, histone lysine methyltransferase and demethylase. Results: The immunoreactivity of EMT-TFs such as Slug (15.6% vs. 42.6%, *p* = 0.005), Twist (23.6% vs. 45.9%, *p* = 0.010) and ZEB1 (15.0% vs. 55.9%, *p* = 0.002) was increased in BM compared with that in lung adenocarcinoma. Epigenetic inducers such as H3K4 methyltransferase (MLL4, *p* = 0.018) and H3K36me3 demethylase (UTX, *p* = 0.003) were statistically increased, and epigenetic repressors such as EZH2 (H3K27 methyltransferase, *p* = 0.046) were significantly decreased in BM compared with those in lung adenocarcinoma. The expression of UTX-ZEB1 (*R*^2^ linear = 1.204) and MLL4-Slug (*R*^2^ linear = 0.987) was increased in direct proportion, and EZH2-Twist (*R*^2^ linear = −2.723) decreased in reverse proportion. Conclusions: The results suggest that certain histone lysine methyltransferase/demethylase, such as MLL4, UTX, and EZH2, regulate the expression of EMT-TFs such as Slug, ZEB1, and Twist epigenetically, which may thereby influence cancer metastasis from the lung to the brain.

## 1. Introduction

Lung cancer is known to be responsible for the highest cancer-related mortalities worldwide, and the same is true in Korea [1]. Histopathologically, non-small cell lung cancer (NSCLC) is the most common type of lung cancer, comprising more than 85% of all lung cancers. More than 70% of patients cannot undergo successful surgical resection because of advanced diseases, such as stage IIIB and IV NSCLC, at initial diagnosis. Although our understanding of NSCLC, and the consequent development of advanced therapeutic strategies for improving the survival of NSCLC patients with further knowledge on the cell-signaling pathway involved, the treatment outcomes are still poor, with a 5-year survival rate of less than 10% in patients with locally advanced or metastatic disease [2]. Patients with lung cancers frequently experience relapse, with metastasis of the disease, even in patients with complete resection of NSCLC. Recently, there have been several reports suggesting that new chemotherapeutic agents such as molecular-targeted therapy, including epidermal growth factor receptor (EGFR) inhibitors and tyrosine kinase inhibitors (TKIs), as well as immunotherapy including anti-PD1 and anti-PDL therapy, can improve the overall survival of the NSCLC patients. However, most patients ultimately develop drug resistance and relapse, despite dramatic initial responses to such treatments. As a result, a progressive disease usually metastasizes to other organs, including the brain, which can negatively affect patients. With brain metastasis (BM), neurological sequelae commonly prevent patients from performing routine daily activities. This decreased engagement in daily activities prevents patients from pursuing additional cancer treatment following the initial poor treatment response and consequent poor prognosis [3]. BM is common in NSCLC patients and is present in 10–20% of NSCLC patients at diagnosis [4]. Approximately 30–50% of NSCLC patients also develop BM during the course of their disease [5]. Patients with BM commonly have a poor prognosis, and a median survival of just one to two months without treatment, mainly due to impaired performance status [3].

The spreading and movement of cancer cells are complicated processes. Among these steps, the epithelial-mesenchymal transition (EMT) is the most important step in the progression of cancers to metastasis and invasion. In cancer cells, which are separated from the original mass of cancerous cells, the process of EMT is activated, facilitating intravasation into the bloodstream [6,7,8,9]. The process of EMT can be aggravated or suppressed at individual steps of genetic or epigenetic expression. The transcriptional drivers and suppressors of the EMT are affected by epigenetic changes such as histone modification and/or the activities of multiple miRNAs [9,10]. Several EMT-associated transcription factors (EMT-TFs) previously studied focused on epigenetic regulation by histone methylation or demethylation [11,12,13,14]. However, these studies have not been performed using human samples, but in vitro, utilizing a culture of stem cells, or in vivo, using a mouse model [15,16], and there are few comprehensive analyses of EMT-TFs that can be regulated by histone modification in human cancer samples. Thus, there are frequent debates as to whether the EMT of cancer cells is truly relevant to the human body, as shown in vivo. EMT is one of the major fields of cancer research; however, the association of the EMT in cancer progression with the outcomes of the treatment has not yet been established. There are many limitations associated with the application of modification of the EMT in clinical practice because an understanding of the role of the EMT in the diagnosis and prediction of therapeutic outcome is still incomplete, despite numerous studies showing that the EMT plays a role in cancer progression. There are many discrepancies in vitro data from real patients originating from the intrinsic heterogeneity of cancer cells and their microenvironments.

Epigenetic events are defined as stable changes in gene expression occurring after transcription, without the alteration of DNA sequences. Epigenetic modifications are thought to play a role in many aspects of health and disease, including cancer biology. There are three major types of epigenetic events; histone modification, RNA interference, and DNA methylation. Histone modification is a major part of the mechanism that post-transcriptionally regulates gene expression. This modification can involve the methylation, acetylation, ubiquitylation, or phosphorylation of histone proteins. The methylation of histone proteins usually occurs at the side chains of arginine and lysine residues within the N-terminal region. Several enzymes regulating the methylation status of histone H3 lysine residues have been reported to be of pathological and clinical significance in several cancers [17,18,19,20,21]. However, there are few studies focused on the EMT process and investigating the unique functions of epigenetic alterations at histone H3 lysine residues and their relationship with cancer progression.

In this study, we investigated the epigenetic role of histone H3 lysine methylation and demethylation on the expression of EMT transcription factors during the metastasis of lung adenocarcinoma to the brain using immunohistochemical analysis for paired lung and brain cancer samples. The primary aim of this study was to identify the specific histone methyltransferase or demethylase of H3 lysine residues that regulate the expression of certain transcriptional driving factors for EMT, which is involved in the metastasis of lung adenocarcinoma to the brain.

## 2. Results

### 2.1. Patient and Tumor Characteristics

Paired samples and clinical data of 46 patients (30 men and 16 women) from 50 cases of lung adenocarcinoma and BM seen in the period between January 2007 and December 2018 were included in this analysis (Table 1). The mean age of these patients at the time of BM diagnosis was 60.2 years (range 42.6–84.5 years). Thirty-four patients (73.9%) had good performance status (Karnofsky Performance Scale (KPS) ≥ 70) and 12 patients (26.1%) had poor performance status (KPS < 70). Only 8 patients (17.3%) had T1 and T2 cancer stages, but 38 patients (82.7%) had T3 and T4 stages at BM confirmation. In addition, only 2 patients (4.3%) had an N1 cancer stage, but 44 patients had N3 and N4 stages at BM confirmation. Except for the brain, the opposite lung is the most common metastatic site of lung adenocarcinoma (*n* = 28, 60.9%), and the adrenal gland is the next most common metastatic site (*n* = 24, 52.2%) (Table 1). Eighteen patients (39.1%) experienced BMs within 2 months of diagnosis of lung adenocarcinoma, and 28 patients (60.9%) found BMs after 2 months of diagnosis of lung adenocarcinoma. Single BM was confirmed in 32 patients (69.6%), 10 patients (21.7%) had oligo-metastases of the brain, and the other 4 patients (8.7%) had four or more BMs (Table 1). Forty patients (87.0%) underwent adjuvant radiotherapy after brain metastasectomy, but 4 patients (8.7%) did not receive any adjuvant treatment for their BM due to poor general condition. After a diagnosis of lung adenocarcinoma, the OS rate was 19.5 months and ranged from 13.2 months to 25.3 months. However, after brain metastasis, the OS rate decreased to 5.8 months (range, 1.4–8.6 months).

### 2.2. Results of Immunohistochemical Staining

All markers, including epithelial markers, mesenchymal markers, EMT-TFs, histone lysine methyltransferase, and demethylase, were examined by immunohistochemical staining (Table 2) and categorized as high immunoreactivity and low immunoreactivity according to the cutoff value, which was determined by receiver operating characteristic (ROC) curve analysis (Table 3).

**Figure 1:**
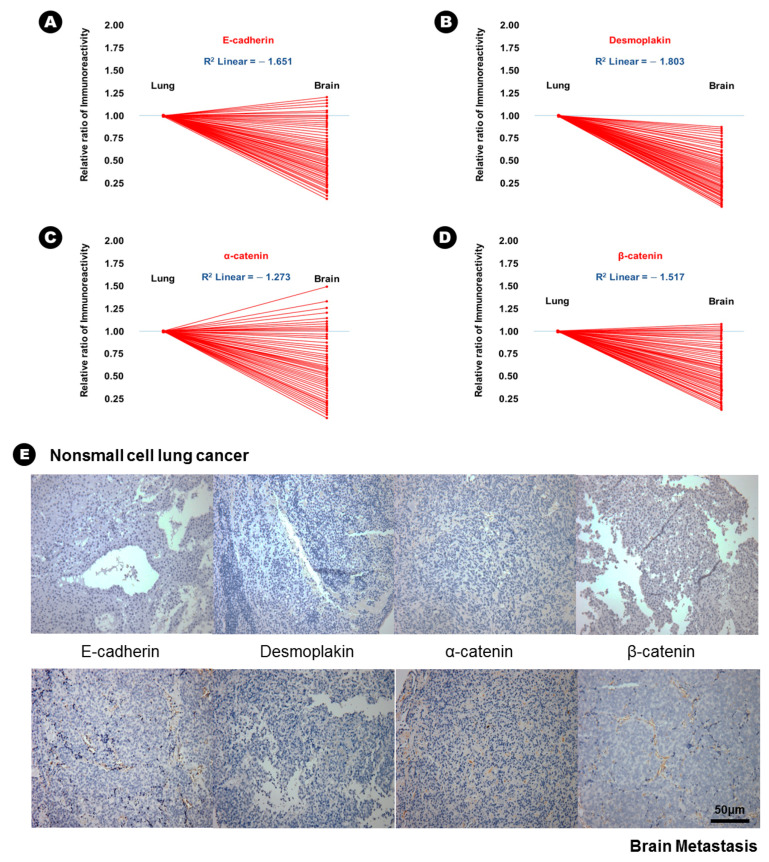
The results of immunohistochemical staining and the relatively decreased patterns of immunoreactivity of epithelial markers from lung adenocarcinoma to brain metastasis. (**A**) E-cadherin, (**B**) desmoplakin, (**C**) α-catenin, (**D**) β-catenin, (**E**) At upper lane, the immunohistochemical staining of epithelial markers of nonsmall cell lung cancer, and those of brain metastasis at bottom lane.

**Figure 2:**
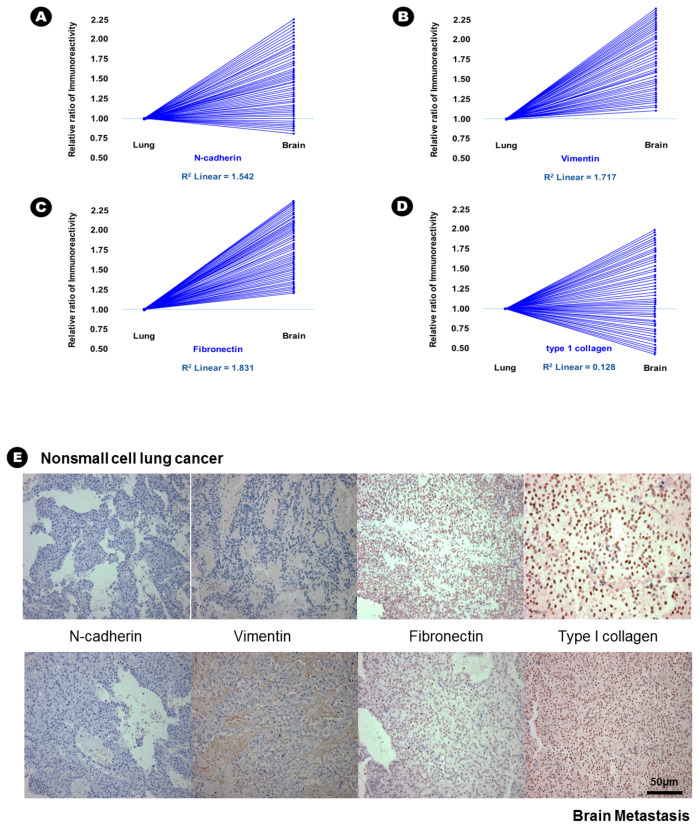
The results of immunohistochemical staining and relative increased patterns of immunoreactivity of mesenchymal markers from lung adenocarcinoma to brain metastasis. (**A**) N-cadherin; (**B**) vimentin and (**C**) fibronectin. An example without a change in immunoreactivity of mesenchymal markers from lung adenocarcinoma to the brain metastasis; (**D**) type 1 collagen; (**E**) At upper lane, the immunohistochemical staining of mesenchymal markers of nonsmall cell lung cancer, and those of brain metastasis at bottom lane.

In terms of epithelial markers, there was a statistically significant decrease in the immunoreactivity of E-cadherin (24.6% vs. 12.6%, *p* = 0.037), desmoplakin (15.6% vs. 2.3%, *p* = 0.007), α-catenin (41.3% vs. 28.3%, *p* = 0.042), and β-catenin (38.6% vs. 16.9%, *p* = 0.029) in BM compared with that in lung adenocarcinoma (Table 2), and an inverse proportion was found in the linear correlation of these markers (Figure 1); this finding indicated that the expression of these four epithelial markers was decreased in BM. Conversely, the immunoreactivity of three mesenchymal markers, N-cadherin (20.6% vs. 43.2%, *p* = 0.028), vimentin (15.3% vs. 51.6%, *p* = 0.004), and fibronectin (7.6% vs. 39.4%, *p* = 0.002) significantly increased in BM compared with that in lung adenocarcinoma (Table 2), and a direct proportion was found in the linear correlation of these markers (Figure 2); this finding indicated that the expression of these three mesenchymal markers was increased in BM.

**Figure 3:**
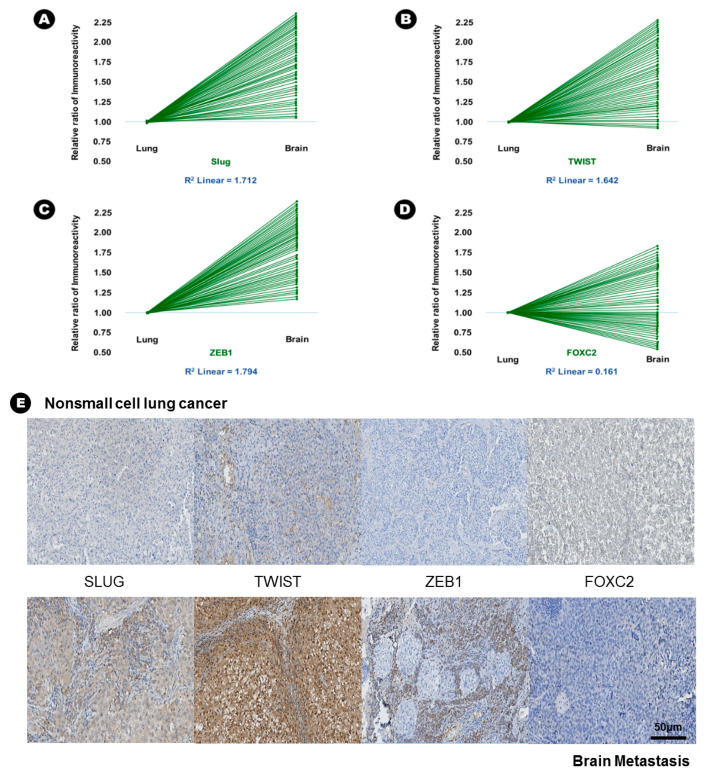
The results of immunohistochemical staining and relative increased patterns of immunoreactivity of an epithelial-to-mesenchymal transition associated transcription factor (EMT-TFs) from lung adenocarcinoma to brain metastasis. (**A**) Slug, (**B**) Twist, and (**C**) ZEB1. An example without a change in immunoreactivity of EMT-TF from lung adenocarcinoma to the brain metastasis: (**D**) FOXC2, (**E**) At upper lane, the immunohistochemical staining of EMT-TFs of nonsmall cell lung carcinoma, and those of brain metastasis at bottom lane.

In terms of EMT-TFs, the immunoreactivity of Slug (15.6% vs. 42.6%, *p* = 0.005), Twist (23.6% vs. 45.9%, *p* = 0.010), and ZEB1 (15.0% vs. 55.9%, *p* = 0.002) was significantly increased in BM compared with lung adenocarcinoma (Table 2), and direct proportion was found in the linear correlation of these markers (Figure 3); this finding indicated that the expression of these three EMT-TFs was increased in BM. Two histone lysine modification enzymes, such as MLL4 (13.9% vs. 46.9%, *p* = 0.018) and UTX (32.4% vs. 70.6%, *p* = 0.003), showed increased immunoreactivity in BM compared with lung adenocarcinoma, indicating that the expression of MLL4 and UTX was increased in BM (Table 2). However, another histone lysine modification enzyme, EZH2 (25.6% vs. 8.6%, *p* = 0.046), showed decreased immunoreactivity in BM compared with lung adenocarcinoma, indicating that the expression of MLL4 and UTX was decreased in BM (Table 2).

### 2.3. Interpretation of the Relationship between EMT-TFs and Histone Lysine Modification

**Figure 4:**
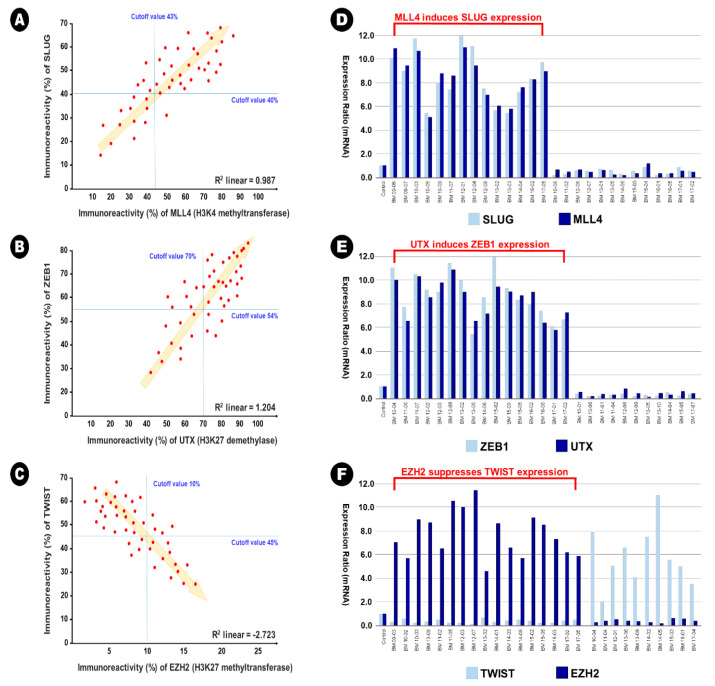
The illustration of epigenetic regulation on the expression between epithelial-to-mesenchymal transition transcription factor and histone lysine modification enzymes in the brain metastasis. (**A**,**D**) MLL4 as H3K4 methyltransferase induces the expression of Slug, (**B**,**E**) UTX as H3K27me3 demethylase induces the expression of ZEB1, and (**C**,**F**) EZH2 as H3H27 demethylase reduces the expression of Twist.

In BM samples, 34 samples (73.9%) showed increased expression of Slug in the immunohistochemical analysis (cutoff value of 40%), and of these samples, 30 samples (88.2%) showed increased expression of MLL4 in the immunohistochemical analysis simultaneously (cutoff value of 43%). A significant linear correlation between Slug expression and MLL4 was also observed in direct proportion (*R*^2^ linear = 0.987) (Figure 4A). This result indicates that MLL4, as an H3K4 methyltransferase, could epigenetically induce Slug expression. In addition, 32 samples (69.6%) showed increased expression of ZEB1 in the immunohistochemical analysis (cutoff value of 54%), and of these samples, 26 samples (81.3%) showed increased expression of UTX in the immunohistochemical analysis simultaneously (cutoff value of 70%). A significant linear correlation between the expression of ZEB1 and UTX was also observed in direct proportion (*R*^2^ linear = 1.204) (Figure 4B). This result indicates that UTX, as an H3K27me3 demethylase, could epigenetically induce the expression of ZEB1. In contrast, 18 samples (78.3%) showed increased expression of Twist in the immunohistochemical analysis (cutoff value of 45%), and of these samples, 17 samples (94.4%) showed decreased expression of EZH2 in the immunohistochemical analysis (cutoff value of 10%). A significant linear correlation between the expression of Twist and EZH2 was also observed in reverse proportion (*R*^2^ linear = −2.723) (Figure 4C). This result indicates that EZH2, as an H3K27 methyltransferase, could epigenetically suppress the expression of Twist. However, these epigenetic relationships were not observed in lung adenocarcinoma samples; *R*^2^ linear of 0.273 between Slug and MLL4, *R^2^* linear of 0.351 between ZEB1 and UTX, and *R*^2^ linear of −0.184 between Twist and EZH2. In addition, no additional statistical correlation was found between other EMT-TFs and histone lysine modifying enzymes in lung adenocarcinoma and BM samples.

These relationships were also found using qRT-PCR. In the samples with simultaneously increased expression of Slug and MLL4, the mean relative ratio in the expression of Slug mRNA was 9.0 (range 5.4~12.0), and that of MLL4 mRNA was 8.6 (range 5.1~10.8) (Figure 4D). In the samples with simultaneously increased expression of ZEB1 and UTX, the mean relative ratio in the expression of ZEB1 mRNA was 8.4 (range 5.6~12.0), and that of UTX mRNA was 8.1 (range 5.5~11.5) (Figure 4E). In the samples with reverse expression of Twist and EZH2, the mean relative ratio in the expression of Twist mRNA was 0.4 (range 0.1~0.8), and that of MLL4 mRNA was 7.7 (range 4.5~11.3) (Figure 4F).

### 2.4. Univariate Analysis of Factors Predicting Survival

In lung adenocarcinoma samples, univariate analysis showed that the high immunoreactivity of two epithelial markers, E-cadherin (hazard ratio (HR), 4.32; 95% confidence interval (CI), 2.46–6.18) and desmoplakin (HR, 3.94; 95% CI, 2.22–5.67); one mesenchymal marker, vimentin (HR, 0.46; 95% CI, 0.25–0.67); and three EMT-TFs, Slug (HR, 0.69; 95% CI, 0.42–0.95), Twist (HR, 0.53; 95% CI, 0.24–0.83) and ZEB1 (HR, 0.59; 95% CI, 0.31–0.88), were associated with longer survival (Table 4).

In BM samples, univariate analysis showed that high immunoreactivity of three mesenchymal markers, N-cadherin (HR, 0.54; 95% CI, 0.15–0.93), vimentin (HR, 0.47; 95% CI, 0.10–0.84), and fibronectin (HR, 0.57; 95% CI, 0.25–0.88), and five EMT-TFs, Snail (HR, 0.58; 95% CI, 0.25–0.92), Slug (HR, 0.41; 95% CI, 0.18–0.65), Twist (HR, 0.26; 95% CI, 0.08–0.44), ZEB1 (HR, 0.19; 95% CI, 0.05–0.53), and FOXC2 (HR, 0.44; 95% CI, 0.35–0.53), were associated with longer survival (Table 5). However, there was no histone lysine modification enzyme predicting longer survival in the univariate analysis of both lung adenocarcinoma and BM.

### 2.5. Multivariate Analysis of Factors Predicting Survival

In terms of clinical factors, the Karnofsky performance scale (KPS) was the sole independent factor for predicting longer survival (HR, 2.772; 95% CI, 1.194–4.779) (Table 6). In lung adenocarcinoma samples, multivariate analysis showed that the following factors were independently associated with longer survival: high expression of E-cadherin (HR, 2.756; 95% CI, 1.347–4.165), low expression of vimentin (HR, 2.627; 95% CI, 1.158–4.096), low expression of Slug (HR, 3.241; 95% CI, 1.873–4.609), and low expression of Twist (HR, 2.976; 95% CI, 1.882–4.071) (Table 6). In BM samples, multivariate analysis showed that the following factors were independently associated with longer survival: low expression of N-cadherin (HR, 3.054; 95% CI, 1.992–4.116), low expression of vimentin (HR, 4.274; 95% CI, 2.607–5.941), low expression of Slug (HR, 3.547; 95% CI, 2.844–4.251), low expression of Twist (HR, 3.913; 95% CI, 3.007–4.819), and low expression of ZEB1 (HR, 2.945; 95% CI, 1.523–4.367) (Table 6).

Additional variables of interest to investigators, such as MLL4, UTX, and EZH2, were examined by multivariate analysis using the Cox regression model. However, these factors were not independently associated with survival (Table 6).

## 3. Discussion

The aim of this study was to evaluate the epigenetic role of histone lysine modification enzymes in regulating the expression of EMT-TFs in lung adenocarcinoma during metastasis to the brain using immunohistochemical analysis of paired human of lung adenocarcinoma and BM samples. To the best of our knowledge, this study is the largest work of translational research analyzing paired samples of human patients to assess the epigenetic role of histone modification enzymes in the regulation of EMT in the metastasis of lung adenocarcinoma to the brain. Although unique histone lysine modification enzymes did not influence the survival of patients directly, several enzymes such as MLL4 (H3K4 methyltransferase), UTX (H3K27me3 demethylase), and EZH2 (H3K27 methyltransferase) regulate the expression of specific EMT-TFs; these EMT-TFs are associated with survival of patients with lung adenocarcinoma via epigenetic regulation. To date, several studies have demonstrated the epigenetic regulation of histone methylation or demethylation of EMT-TFs. However, these studies have not been performed on human samples, and there are few comprehensive analyses of EMT-TFs that can be regulated by histone modification in human cancer samples. For example, in breast cancer, there are several studies that have shown the epigenetic role of histone modification of EMT-TF expression in the metastasis process. The recruitment of histone lysine-specific demethylase 1 (LSD1) to the E-cadherin promoter for the demethylation of histone H3 lysine 4 (H3K4) is reported to bridge H3K4 demethylation to DNA methylation on the E-cadherin promoter [11]. In addition, one of the EMT-TFs, Snail, interacted with H3K9 methyltransferase G9a and Suv39H1, the two major methyltransferases responsible for H3K9 methylation that are intimately linked to DNA methylation [22]. These epigenetic roles of histone lysine modifying enzymes could explain the detailed mechanism underlying E-cadherin silencing in breast cancer. EZH2 has also been suggested to promote metastasis through pleiotropic roles in modifying EMT, such as repression of tumor suppressor genes or miRNA, as well as regulation of cancer stem cells and migration [23].

The full mechanism of epigenetic regulation of the EMT process via histone lysine modification cannot be understood without a definition of the EMT. Such a description must include the unique genetic alterations that may occur during the transition from an epithelial phenotype to a mesenchymal phenotype, resulting in the capacity for metastasis. There have been many in vitro experiments showing successful results from changes in the expression of epithelial and mesenchymal biomarkers, suggesting that these molecules play a role in the EMT process [24]. However, these results have not been reproducible or validated in vivo or in clinical practice. Therefore, there is still some confusion and much debate on the role of genetic and epigenetic alterations in the EMT process during cancer metastasis [25]. To overcome these limitations, cells bearing the hybrid phenotype such as circulating tumor cells (CTCs) have been studied. The present study could not show any results of serial cell lineage from EMT to mesenchymal-to-epithelial transition (MET) using CTC. However, there have been several efforts to define the role of the EMT and the MET process without a comprehensive study using serial cells from primary cancer to metastasis in the clinical fields. Xia et al. [20] suggested that a quantitative EMT scoring system based on the genetic expression profile of the primary cancer, scoring the state of EMT from −1.0 to +1.0, could define the steps of metastasis. These authors showed that this system could identify the specific characteristics of the steps of the EMT process in the individual pathology of the cancers [20]. The importance of identifying the individual steps of the EMT process in predicting patient prognosis and the clinical course has also been reported [20,26]. Comprehensive information about the regulation of the EMT process could provide the advanced ability to predict the clinical outcome of cancer patients. Despite the absence of serial analysis using CTC, the changes in the expression pattern of EMT-associated markers, including epithelial and mesenchymal markers, as well as transcriptional markers, in primary cancer cells and their metastatic counterparts can help investigators define the likelihood of the development of EMT. If there are phenotypes intermediate between primary cancer and metastatic tumors, it is possible to identify the specific cells in the stromal compartment involved due to the tendency of the cancers to maintain the epithelial characteristics of their tissue of origin [6].

According to the widest study for predicting the prognosis of BM patients, the following factors are associated with the prognosis of patients with BM of lung adenocarcinoma; age, KPS, extracranial metastasis, and number of BMs [27,28]. However, the present study showed that the sole factor of KPS was statistically associated with survival in multivariate analysis. Because of the limited selection, our subjects were patients who underwent surgical resection for BM. Therefore, the number of patients was too small to reflect the whole cohort with BM of lung adenocarcinoma, which limits the applications of the data to clinical practice.

Although our study suggests a meaningful role for several histone lysine modification enzymes, such as UTX, MLL4, and EZH2, in regulating the expression of EMT-TFs during the metastasis of lung adenocarcinoma to the brain, it has several limitations. First, our analyses in this study were performed using only immunohistochemical staining and qRT-PCR to define the epigenetic roles of UTX, MLL4, and EZH2 on the regulation of the expression of EMT-TFs. However, UTX is just one of several mechanisms demethylating H3K36m3, MLL4 is one of several mechanisms methylating H3K4, and EZH2 is also one of several mechanisms H3K27. They cannot represent the function of methylation of H3K36me, H3K4m1/2/3 and H3K27m1/2/3, respectively, in cancer biology. In terms of EZH2, mutation or overexpression of EZH2 is known to help cancerous cells divide and proliferate by inhibiting genes responsible for suppressing tumor development. EZH2 is an attractive target for anti-cancer therapy because it is upregulated in multiple cancers, including, but not limited to, breast [29], prostate [30], melanoma [31], bladder cancer [32], and lymphoma [33]. However, the role of EZH2 has not been comprehensively established in lung adenocarcinoma. The major role of EZH2 in oncogenesis is known to engage in the proliferation and development rather than the EMT process of the cancer [34]. Actually, Abdel et al. [35] reported that the expression of Twist and EZH2 was significantly higher in colon cancer than that in the normal colonic mucosa and suggested that Twist and EZH2 should serve as prognostic predictors for colon cancer, respectively. However, they did not show the relationship or interaction of Twist with EZH2. In our study, the immunoreactivity of Twist (23.6% vs. 3.3%; *p* = 0.013) and EZH2 (25.6% vs. 8.2%; *p* = 0.037) was also significantly higher in lung adenocarcinoma than in epithelial cell of the normal lung. However, the immunoreactivity of Twist (45.9% vs. 2.7%; *p* = 0.005) was still significantly higher in brain metastasis than in the normal brain, but that of EZH2 (8.6% vs. 7.8%; *p* = 0.834) was not different between brain metastasis and normal brain. Briefly, the EZH2 expression was not significantly increased in brain metastasis, unlike their primary cancer (lung adenocarcinoma), nor was it associated with the prognosis of lung adenocarcinoma patients independently. This discrepancy may be originated from the different expressions between lung adenocarcinoma and brain metastasis, the different cancer cell type, and the different sample size. As the traditional oncogenic function of EZH2 is focused on inducer of cancer cell proliferation and development, further comprehensive research is necessary for defining the other role of EZH2, such as EMT process, invasion, and angiogenesis. Although the present study suggested that these histone modifications influence the metastasis of lung adenocarcinoma to the brain, more comprehensive scientific evidence, supported by molecular genetic analysis using in vivo as well as in vitro study, is needed to validate the present results. It is also important to identify the unique target genes of these histone lysine modification enzymes to determine their role in the metastasis of lung adenocarcinoma to the brain.

Second, although two different neuropathologists assessed immunoreactivity in the samples, it is not certain whether our assessment of experiments in this study should be absolutely correct or not because the interpretation of the results obtained by immunohistochemical staining may be rather subjective. The optimal assessment of immunohistochemical staining results can differ according to the concentration of the antigen used for staining because of the difficulty in establishing standard conditions. In addition, there is no standard rule for determining the cutoff value between positive and negative findings. Therefore, it is necessary to establish a reasonable cutoff value in order to repeat the experiments for validation and to cooperate and communicate detail regarding the interpretation of the data among the investigators. In order to overcome the flaws in immunohistochemical staining, we used ROC curve analysis to establish the cutoff value in a principled manner. To determine the identity of immunoreactivity for the EMT and MET markers, EMT-TFs, and histone lysine modification enzymes at the cell level, an in vitro study will be helpful. Recently, a deep-learning-based method using artificial intelligence that can automatically localize and quantify the regions expressing biomarker in any selected area on a whole slide image is proposed [36].

The third limitation is the lack of examination of all EMT markers, MET markers, EMT-TFs, and histone lysine modification enzymes that may be implicated. Importantly, there are many EMT-TF families, such as Snail (zinc finger proteins Snail and Slug), Zeb (zinc finger and homeodomain proteins Zeb1 and Zeb2), and Twist (basic helix-loop-helix proteins E12, E47, Twist1, Twist2, and Id), which have important functions in the normal development of human and EMT processes associated with cancer biology. These EMT-TFs are known to be major drivers of the EMT [37]. They can induce the dedifferentiation process of the epithelial component of cancer cells by repressing the transcription of epithelial gene transcription such as E-cadherin, known as a classic epithelial expressing gene; and activating mesenchymal gene such as N-cadherin as a classic mesenchymal expressing gene [37,38] However, we did not analyze all EMT-TFs; therefore, our results cannot reflect all the possible mechanisms of epigenetic regulation of EMT in lung adenocarcinomas. It is necessary for the investigators to perform a sequencing analysis to determine the target genes in human samples and validate the results with in vivo and in vitro studies.

Finally, another limitation of this study is the bias originating from the retrospective design of the study. If the sample size is sufficiently large, it can surmount this obstacle. However, our study involved a relatively small number of subjects and may, therefore, not meet the assumptions of the statistical tests used. We did our best to reduce the bias by obtaining the clinical data obtained from computerized data archives using a uniform system and included the candidate patients who were treated with the same protocol in a single center. The multiple researchers involved in this study did not have any clinical information or experimental results to help avoid preconception. We also independently reviewed the pathological slides and radiological images, and we cannot clearly say that no bias originated from this retrospective study. Despite these efforts, however, the conclusions drawn from our study need further validation through prospective and randomized clinical trials.

## 4. Materials and Methods

### 4.1. Sample Collection

We conducted a translational cohort study using formalin-fixed, paraffin-embedded (FFPE) tissue specimens from 46 patients, all of which were paired samples of lung adenocarcinoma and its respective BM. A clinical review of patients with lung adenocarcinoma and BM was performed. All patients underwent biopsy or lobectomy of lung adenocarcinoma and biopsy or surgical resection for BM and were histopathologically confirmed at our institute from January 2007 to December 2018. During this period, 527 patients were histopathologically diagnosed with lung adenocarcinoma through biopsy or lobectomy, and 96 patients (18.2%) were radiographically diagnosed with BM of lung adenocarcinoma at our institute. Among the 96 patients with BM, 50 (52.1%) underwent histopathological diagnosis (biopsy in 8 patients (16.0%) and craniotomy with curative resection of BMs in 42 patients (84.0%)). All patients included in this study had a newly diagnosed lung adenocarcinoma and its BM and received treatment and follow-up at our institution until death. Our institute is the sole regional university hospital serving a population of 1,500,000 people. The available histological samples were obtained from the Department of Pathology Archives of our institute. All hematoxylin and eosin-stained slides were reviewed by two pathologists using the 2015 WHO classification of lung tumors and were blinded to the clinical and pathological parameters [39]. Four samples (8.0%) were excluded because the tissue was almost entirely necrotized, or the tumor contribution to the section was less than 80%. In total, data from 46 patients were included in this study.

### 4.2. Clinical and Radiological Data

Epidemiological characteristics (including gender, age at the time of diagnosis of lung adenocarcinoma, KPS score, and timing of metastasis to the brain), type of primary treatment for lung adenocarcinoma, type of salvage treatment for BM, length of follow-up, and time of death were retrospectively reviewed in the medical records of each patient. In terms of the timing of BM from lung adenocarcinoma, BM diagnosed ≤2 months from lung adenocarcinoma diagnosis was referred to as synchronous metastasis and diagnosed >2 months from the time of lung adenocarcinoma diagnosis was described as metachronous metastasis.

Clinical T and N stages were estimated by abdominal and chest computed tomography (CT) as well as positron emission tomography (PET)-CT performed at the time of diagnosis of lung adenocarcinoma. The metastatic sites were examined through both the intra- and extra-thoracic cavities. The number of BM was classified as the mass enhanced with gadolinium in the T1 weighted magnetic resonance image (MRI). Radiological evaluation was performed by two different neuroradiologists who were blinded to the clinical and pathological parameters.

In terms of treatment, the radiation dosage, type of radiotherapy administered, and regimen and timing of chemotherapy were examined. Clinical indications for surgical resection of BM included symptoms and signs of intracranial hypertension unresponsive to adequate medical therapy (e.g., corticosteroid and mannitol), intractable seizures, reduced level of consciousness, progressive motor weakness, gait ataxia, or aphasia. Patients with the following clinical indications were also considered as candidates for surgical resection: growing brain lesion, associated hemorrhage, and a mass effect due to edema unresponsive to maximal medication in the MRI. A stereotactic biopsy for the brain lesion was considered when the radiological diagnosis was indeterminate for BM.

### 4.3. Immunohistochemical Staining and Its Interpretation

With immunohistochemical staining, all tissue specimens were examined for the expression of epithelial markers (E-cadherin, occludins, desmoplakin, α-catenin, β-catenin, and type IV collagen); mesenchymal markers (N-cadherin, vimentin, fibronectin, α5β1 integrin, αγβ6 integrin, and type I collagen); EMT-TFs (SNAIL, Slug, Twist, ZEB1, and FOXC2); histone lysine methyltransferase (myeloid/lymphoid or mixed-lineage leukemia 4 (MLL4); retinoblastoma interacting zinc finger (RIZ); enhancer of zester homolog 2 (EZH2); nuclear receptor binding SET domain protein 2 (NSD2)); histone lysine demethylase (lysine-specific histone demethylase 1A (LSD1); Jumonji domain-containing 1 (JMJD1); ubiquitously transcribed tetratricopeptide repeat X chromosome (UTX); and Jumonji domain-containing 5 (JMJD5)). We obtained three or four sections sequentially from each FFPE block of lung adenocarcinoma and BM per patient. For immunohistochemical analysis, the labeled streptavidin-biotin method was applied to sections from FFPE tissues that had been used for disease diagnosis. Individual monoclonal or polyclonal primary antibodies were used according to the manufacturer’s instructions (Table S1).

Appropriate positive and negative immunohistochemical controls were used throughout the study. Negative controls were samples in which the primary antibody had been omitted. Sections from the normal brain cortex obtained from autopsy specimens were used as positive controls for detecting each marker. Ten fields were selected from the regions with the highest concentrations of immunohistochemically stained nuclei and were examined at high-power magnification (×400). Each field corresponded to a total cell number ranging from 700 to 1000 cells relative to the cellularity of the tumor specimen and areas of necrosis; normal glial cells, normal epithelial cells of the lung, and endothelial cells were excluded. Considering 1000 cells by manual counting, we recorded the immunoreactivity of proteins and markers as the percentage of immunohistochemically stained cells.

Two different neuropathologists (E.H. Lee and D.C. Kim), who were both blinded to patient clinical and radiological information, reviewed all slides. If the difference between the percentages of immunoreactive cells calculated independently by the two pathologists was less than 5%, the mean of the two percentages was used. If the difference was 5% or more, defined as discordance, two reviewers determined the mean percentage of immunoreactivity after repeatedly counting the cells, and there was only one discordant case (4.3%) of immunoreactivity. Digital images were captured with a microscope (model BX41TF, Olympus, Tokyo, Japan) and a digital camera (model DP70, Olympus, Tokyo, Japan).

As there is no universal cutoff value for the immunoreactivity of these markers, the area under the ROC curve was used to determine the optimal threshold of the mean percentage of immunoreactive cells from 1000 cells. Sensitivity was calculated as the true positive rate (the number of true positives divided by the sum of the numbers of true positives and false negatives), specificity was estimated as the true negative rate (the number of true negatives divided by the sum of the numbers of true negatives and false positives), and accuracy as the sum of the number of true positives and true negatives divided by the total number of positives and negatives [40]. True positives were those in which the immunoreactivity percentage above the cutoff value had a positive influence on overall survival (OS), and true negatives, in which the immunoreactivity percentage below the cutoff value had a negative influence on OS. We determined the threshold of immunoreactivity with the greatest sensitivity and specificity. Through a sensitivity-specificity analysis, a cutoff point for immunoreactivity at which sensitivity and specificity crossed and that was correlated with longer survival was determined for each marker. According to the cutoff value established for these markers, sequential correlation analysis for OS among the patients was performed.

### 4.4. Quantitative Reverse Transcription Polymerase Chain Reaction for mRNA

For RNA extraction, seven serial sections of 8–10 micrometer thickness per sample were taken using a standard microtome (Leica SM2000 R Sliding Microtome, Wetzlar, Germany) with disposable blades. The sections were collected directly into sterile 1.5 mL microcentrifuge tubes. The sections in tubes were deparaffinized with two prewarmed xylene washes followed by 95%, 75%, and 50% ethanol rinses as previously described [41]. Briefly, the tissue pellets were dried at 37 °C. All pellets were digested with 20 μL proteinase K (20 mg/mL proteinase K, Roche Diagnostics GmbH, Mannheim, Germany) and 180 μL digestion buffer (10 mM Tris-HCl, pH 8.0.100 mM EDTA, pH 8.0.50 mM NaCl, and 0.5% SDS). Total RNA was isolated using TRIzol reagent (Life Technologies, Carlsbad, CA, USA) according to the manufacturer’s instructions, and RNA concentration was determined using a spectrophotometer. For mRNA quantitation, reverse transcription into complementary DNA (cDNA) was performed using a high-capacity cDNA reverse transcription kit (0049472, Thermo Fisher, Waltham, MA, USA) following the manufacturer’s instructions. Power SYBR™ Green PCR Master Mix (4367659, Thermo Fisher, Waltham, MA, USA) was used for qRT-PCR with a total reaction volume of 20 μL. GAPDH was used as an internal control. All primers were synthesized by OriGene Technologies, Inc. (Rockville, MD, USA) (Table S2). The mRNA levels were determined using Quant Studio 3 and 5 Systems (Applied Biosystems, Foster City, CA, USA). For relative quantitation, expression levels were calculated using CT values (corrected for GAPDH expression) according to the equation: 2^−^^∆CT^ [∆CT = CT (gene of interest) − CT (GAPDH)]. All qRT-PCR analyses were performed in triplicates. The genes of EMT-associated transcriptional factors and histone-modifying enzymes were estimated by quantitative reverse transcription polymerase chain reaction (qRT-PCR) in order to validate the results of immunohistochemical staining.

### 4.5. Survival Analysis and Statistical Analysis

Medical records of clinical history and radiographic reports of all study subjects were analyzed. The date of death was confirmed and recorded. OS was defined as the time from the date of diagnosis of lung adenocarcinoma and BM until death. The date of biopsy or surgical resection of lung adenocarcinoma was recorded as the date of diagnosis.

Statistical analyses were performed using SPSS ver. 20.0 (IBM Corp., Armonk, NY, USA). Differences between subgroups were analyzed with Student’s *t*-test for normally distributed continuous values, Mann–Whitney U test for non-normally distributed continuous values, and chi-squared tests were used to analyze categorical variables. OS was calculated according to the Kaplan–Meier method. Comparisons among groups were performed using log-rank tests. Variables that were significantly associated with longer OS of lung adenocarcinoma patients with BM in univariate analyses were examined using multivariate analysis. Several additional variables associated with OS in the literature and of interest to investigators were also included in the multivariate analysis. In this analysis, the Cox proportional hazards regression model was used to assess the independent effects of specific factors on OS and define hazard ratios of significant covariates. Two-sided *p* values < 0.05 were considered statistically significant.

### 4.6. Ethical Statement

The Institutional Review Board (IRB) of our institute approved the study protocol (IRB number: SCMC 2016-12-005) on 23 February 2017. It is declared that this study should be conducted according to the guidelines of the Declaration of Helsinki for biomedical research. Written informed consent was waived due to its retrospective nature.

## 5. Conclusions

In this study, we investigated the epigenetic role of histone H3 lysine modification in the regulation of EMT transcriptional driving factors expression during the metastasis of lung adenocarcinoma to the brain using immunohistochemical analysis of the paired cancer samples from the lung and brain of patients. We found that MLL4 (H3K4 methyltransferase), UTX (H3K27me3 demethylase), and EZH2 (H3K27 methyltransferase) regulate the post-transcriptional expression of EMT-TFs Slug, Twist, and ZEB1, respectively. Through this epigenetic regulation by histone lysine modification, Slug, Twist, and ZEB1 is likely associated with longer survival in patients with BM of lung adenocarcinoma. Application of the results of this study to research and clinical trials can facilitate the development of new treatments.

## Figures and Tables

**Table 1:** Clinical features of entire cohorts with brain metastasis of lung adenocarcinoma at the time of diagnosis of metastasis to the brain (*n* = 46).

Clinical Features		Number (%)
**Gender**	Male	30 (65.2%)
	Female	16 (34.8%)
Mean Age (Year)		60.2 (42.6–84.5)
KPS	≥70	34 (73.9%)
	<70	12 (26.1%)
Clinical T Stage	T1	2 (4.3%)
	T2	6 (13.0%)
	T3	20 (43.6%)
	T4	18 (39.1%)
Clinical N Stage	N0	0 (0.0%)
	N1	2 (4.3%)
	N2	32 (69.6%)
	N3	12 (26.1%)
Timing of Brain Metastasis	Synchronous	18 (39.1%)
	Metachronous	28 (60.9%)
Number of Brain Metastasis	1	32 (69.6%)
	2–3	10 (21.7%)
	≥4	4 (8.7%)
Metastatic Site	Brain	46 (100.0%)
	Adrenal Gland	24 (52.2%)
	Liver	6 (13.0%)
	Bone	18 (39.1%)
	Opposite Lung	28 (60.9%)
	Others	8 (17.4%)
Treatment after Brain Metastasis	Cytotoxic Chemotherapy	10 (21.7%)
	Target Therapy	34 (73.7%)
	Immunotherapy	12 (26.1%)
	Radiotherapy	40 (87.0%)
	Combination	42 (91.3%)
Survival after Diagnosis of Lung Adenocarcinoma (95% CI)		19.5 (13.2–25.3)
Survival after Brain Metastasis (95% CI)		5.8 (1.4–8.6)

Abbreviations: CI, confidence interval; KPS, Karnofsky performance scale.

**Table 2:** Immunohistochemical staining of EMT/MET markers, transcription factors, and histone modification enzymes on the lung adenocarcinoma and brain metastasis in the paired samples (*n* = 46).

Factors	Lung Adenocarcinoma	Brain Metastasis	*p* Value
Epithelial Markers			
E-cadherin	24.6%	12.6%	0.037
Occludins	37.4%	28.9%	0.254
Desmoplakin	15.6%	2.3%	0.007
α-catenin	41.3%	28.3%	0.042
β-catenin	38.6%	16.9%	0.029
Type IV collagen	52.3%	45.3%	0.384
Mesenchymal Markers			
N-cadherin	20.6%	43.2%	0.028
Vimentin	15.3%	51.6%	0.004
Fibronectin	7.6%	39.4%	0.002
α5β1 integrin	16.3%	18.9%	0.843
αγβ6 integrin	22.6%	27.4%	0.726
Type I collagen	37.8%	45.2%	0.662
Transcription Factors			
Snail	38.6%	28.7%	0.154
Slug	15.6%	42.6%	0.005
Twist	23.6%	45.9%	0.010
ZEB1	15.0%	55.9%	0.002
FOXC2	35.6%	47.2%	0.527
Histone Methyltransferase			
MLL4 (H3K4)	13.9%	46.9%	0.018
RIZ (H3K9)	38.6%	33.3%	0.561
EZH2 (H3K27)	25.6%	8.6%	0.046
NSD2 (H3K36)	20.5%	22.8%	0.837
Histone Demethylase			
LSD1 (H3K4)	42.3%	28.9%	0.170
JMJD1 (H3K9)	20.5%	24.3%	0.642
UTX (H3K27)	32.4%	70.6%	0.003
JMJD5 (H3K36)	15.6%	22.1%	0.389

Abbreviation: EMT, epithelial-to-mesenchymal transition; MET, mesenchymal-to-epithelial transition.

**Table 3:** Results of receiver operating characteristic (ROC) curve analysis of EMT/MET markers, transcription factors, and histone modification enzymes determining the cutoff value on the lung adenocarcinoma and the brain metastasis.

Factors	Lung Adenocarcinoma			Brain Metastasis		
	Mean% of IHC Staining Nuclei (%, ± SD)	AUC in ROC Curve	Cutoff Value (%)	Mean% of IHC Staining Nuclei (%, ± SD)	AUC in ROC Curve	Cutoff Value (%)
Epithelial Markers						
E-cadherin	24.6 ± 8.6	0.58	25	12.6 ± 7.2	0.62	14
Occludins	37.4 ± 18.9	0.64	35	28.9 ± 13.5	0.64	30
Desmoplakin	15.6 ± 7.9	0.71	15	2.3 ± 1.2	0.72	2
α-catenin	41.3 ± 24.0	0.68	40	28.3 ±10.8	0.70	28
β-catenin	38.6 ± 17.5	0.72	37	16.9 ± 8.6	0.77	15
Type IV Collagen	52.3 ± 31.9	0.62	50	45.3 ± 22.9	0.72	45
Mesenchymal Markers						
N-cadherin	20.6 ± 11.2	0.71	20	43.2 ±20.7	0.78	41
Vimentin	15.3 ± 8.8	0.69	15	51.6 ± 34.5	0.70	50
Fibronectin	7.6 ± 4.6	0.60	7	39.4 ± 15.6	0.75	38
α5β1 integrin	16.3 ± 7.0	0.73	15	18.9 ± 8.6	0.67	17
αγβ6 integrin	22.6 ± 10.9	0.79	21	27.4 ± 10.5	0.71	25
Type I collagen	37.8 ± 20.1	0.64	36	45.2 ± 22.7	0.68	45
Transcription Factors						
Snail	38.6 ± 16.4	0.84	35	28.7 ± 13.0	0.72	27
Slug	15.6 ± 4.8	0.75	15	42.6 ± 19.4	0.58	40
Twist	23.6 ± 10.8	0.72	22	45.9 ± 26.8	0.68	45
ZEB1	15.0 ± 6.9	0.68	14	55.9 ± 31.3	0.61	54
FOXC2	35.6 ± 17.3	0.67	36	47.2 ± 15.9	0.63	46
Histone Methyltransferase						
MLL4(H3K4)	13.9 ± 6.3	0.75	14	46.9 ± 20.4	0.73	43
RIZ(H3K9)	38.6 ± 14.6	0.80	37	33.3 ± 18.1	0.68	31
EZH2(H3K27)	25.6 ± 15.4	0.67	25	8.6 ± 4.2	0.59	10
NSD2(H3K36)	20.5 ± 9.5	0.74	20	22.8 ± 9.8	0.62	22
Histone Demethylase						
LSD1(H3K4)	42.3 ± 18.6	0.65	41	28.9 ± 11.3	0.64	27
JMJD1(H3K9)	20.5 ± 8.2	0.76	20	24.3 ± 9.2	0.70	25
UTX(H3K27)	32.4 ± 15.0	0.84	34	70.6 ± 27.3	0.69	70
JMJD5(H3K36)	15.6 ± 6.7	0.67	16	22.1 ± 7.6	0.75	22

Abbreviations: AUC, area under the curve; IHC, immunohistochemical; ROC, receiver operating characteristic; SD, standard deviation.

**Table 4:** Univariate analysis of factors predicting longer survival according to the immunoreactivity of the EMT/MET markers and transcription factors on the lung adenocarcinoma.

Immunoreactivity		Overall Survival (Mean, Months)	Hazard Ratio	95% CI	*p* Value
E-cadherin	Low	11.3	1.00	2.46–6.18	0.002
	High	27.0	4.32		
Occludins	Low	18.6	1.00	0.88–1.64	0.724
	High	20.3	1.26		
Desmoplakin	Low	13.7	1.00	2.22–5.67	0.014
	High	24.8	3.94		
α-catenin	Low	17.0	1.00	0.96–3.34	0.062
	High	21.8	2.15		
β-catenin	Low	19.2	1.00	0.64–1.62	0.884
	High	19.8	1.13		
Type IV collagen	Low	20.4	1.00	0.47–1.46	0.903
	High	18.7	0.97		
N-cadherin	Low	21.6	1.00	0.13–1.03	0.067
	High	17.6	0.58		
Vimentin	Low	23.1	1.00	0.25–0.67	0.034
	High	16.2	0.46		
Fibronectin	Low	20.8	1.00	0.55–1.09	0.073
	High	18.3	0.82		
α5β1 integrin	Low	19.8	1.00	0.74–1.22	0.325
	High	19.2	0.98		
αγβ6 integrin	Low	21.3	1.00	0.26–1.08	0.091
	High	17.9	0.67		
Type I collagen	Low	22.0	1.00	0.18–1.09	0.069
	High	17.2	0.63		
Snail	Low	21.6	1.00	0.47–1.15	0.084
	High	17.6	0.81		
Slug	Low	22.9	1.00	0.42–0.95	0.042
	High	16.4	0.69		
Twist	Low	25.1	1.00	0.24–0.83	0.007
	High	14.4	0.53		
ZEB1	Low	24.3	1.00	0.31–0.88	0.010
	High	15.1	0.59		
FOXC2	Low	22.0	1.00	0.27–1.26	0.206
	High	17.2	0.76		

Abbreviations: CI, confidence interval; EMT, epithelial-to-mesenchymal transition; MET, mesenchymal-to-epithelial transition.

**Table 5:** Univariate analysis of factors predicting longer survival according to the immunoreactivity of the EMT/MET markers and transcription factors on the brain metastasis.

Immunoreactivity		Overall Survival (Mean, Months)	Hazard Ratio	95% CI	*p* Value
E-cadherin	Low	20.3	1.00	0.54–1.24	0.416
	High	18.7	0.84		
Occludins	Low	18.6	1.00	0.78–2.26	0.524
	High	20.3	1.52		
Desmoplakin	Low	21.6	1.00	0.36–1.14	0.231
	High	17.6	0.75		
α-catenin	Low	20.4	1.00	0.64–1.18	0.448
	High	18.7	0.91		
β-catenin	Low	19.0	1.00	0.54–1.90	0.643
	High	19.9	1.22		
Type IV collagen	Low	17.2	1.00	0.93–4.77	0.152
	High	21.6	2.85		
N-cadherin	Low	22.3	1.00	0.15–0.93	0.027
	High	16.9	0.54		
Vimentin	Low	25.4	1.00	0.10–0.84	0.004
	High	14.1	0.47		
Fibronectin	Low	23.4	1.00	0.25–0.88	0.020
	High	15.9	0.57		
α5β1 integrin	Low	21.7	1.00	0.34–1.08	0.065
	High	17.5	0.71		
αγβ6 integrin	Low	22.1	1.00	0.42–1.17	0.118
	High	17.1	0.79		
Type I collagen	Low	21.5	1.00	0.48–1.21	0.251
	High	17.7	0.84		
Snail	Low	23.5	1.00	0.25–0.92	0.014
	High	15.8	0.58		
Slug	Low	27.6	1.00	0.18–0.65	0.009
	High	12.1	0.41		
Twist	Low	28.4	1.00	0.08–0.44	0.005
	High	11.4	0.26		
ZEB1	Low	32.1	1.00	0.05–0.34	<0.001
	High	8.1	0.19		
FOXC2	Low	24.6	1.00	0.35–0.53	0.012
	High	14.8	0.44		

Abbreviation. CI, confidence interval; EMT, epithelial-to-mesenchymal transition; MET, mesenchymal-to-epithelial transition.

**Table 6:** Multivariate analysis of factors predicting longer survival according to the immunoreactivity of the EMT/MET markers, transcription factors, and histone modification enzymes on the lung adenocarcinoma and the brain metastasis.

Factors	Hazard Ratio	95% CI	*p* Value
Age (<60 years vs. ≥60 years)	1.820	0.852–2.788	0.275
KPS (≥70 vs. <70)	2.772	1.194–4.779	0.042
Number of BM (≥4 vs. <4)	1.542	0.765–2.319	0.482
Timing of BM (≥2 months vs. <2 months)	1.286	0.684–1.888	0.671
Clinical T Stage (4 vs. 1–3)	2.235	0.917–3.553	0.106
Clinical N Stage (4 vs. 1–3)	2.418	0.943–3.893	0.062
E-cadherin of lung (high vs. low)	2.756	1.347–4.165	0.044
Desmoplakin of lung (high vs. low)	1.368	0.886–1.850	0.442
Vimentin of lung (low vs. high)	2.627	1.158–4.096	0.048
Slug of lung (low vs. high)	3.241	1.873–4.609	0.020
Twist of lung (low vs. high)	2.976	1.882–4.071	0.027
ZEB1 of lung (low vs. high)	2.480	0.935–4.024	0.056
N-cadherin of brain (low vs. high)	3.054	1.992–4.116	0.018
Vimentin of brain (low vs. high)	4.274	2.607–5.941	0.002
Fibronectin of brain (low vs. high)	1.942	0.907–2.978	0.152
Snail of brain (low vs. high)	1.802	0.806–2.798	0.199
Slug of brain (low vs. high)	3.547	2.844–4.251	0.011
Twist of the brain (low vs. high)	3.913	3.007–4.819	0.006
ZEB1 of brain (low vs. high)	2.945	1.523–4.367	0.038
FOXC2 of brain (low vs. high)	2.004	0.897–3.111	0.086
MLL4 of brain (low vs. high)	1.252	0.647–1.857	0.574
EZH2 of brain (high vs. low)	1.674	0.579–2.769	0.306
UTX of brain (low vs. high)	1.842	0.806–2.878	0.155

Abbreviations: BM, brain metastasis; CI, confidence interval; EMT, epithelial-to-mesenchymal transition; KPS, Karnofsky performance scale; MET, mesenchymal-to-epithelial transition.

## Supplementary Materials

The following are available online at https://www.mdpi.com/2072-6694/12/12/3632/s1, Table S1: The lists of primary antibodies for immunohistochemical staining of EMT/MET markers, transcription factors, and histone modification enzymes on the lung adenocarcinoma and brain metastasis in the paired samples, Table S2: The lists of primers for quantitative reverse transcription–polymerase chain reaction for mRNA of the candidate genes of transcription factors associated with epithelial-to-mesenchymal transition, and histone modification enzymes on the lung adenocarcinoma and brain metastasis.
