# Supplementary material for: Epigenetic Role of Histone Lysine Methyltransferase and Demethylase on the Expression of Transcription Factors Associated with the Epithelial-to-Mesenchymal Transition of Lung Adenocarcinoma Metastasis to the Brain
Source: Cancers (Basel). 2020 Dec 4;12(12):3632. doi: 10.3390/cancers12123632 (PMC7761791; doi:10.3390/cancers12123632)
Supplement: Supplementary file 1 [file cancers-12-03632-s001.pdf]

cancers

MDPI

Article

Epigenetic Role of Histone Lysine Methyltransferase and Demethylase on the Expression of Transcription Factors Associated with the Epithelial-to-Mesenchymal Transition of Lung Adenocarcinoma Metastasis to the Brain

Young Min Lee, Seok Hyun Kim, Minseok S. Kim, Dae Cheol Kim, Eun Hee Lee, Ju Suk Lee, Sung-Hun Lee and Young Zoon Kim

Table S1. The lists of primary antibodies for immunohistochemical staining of EMT/MET markers, transcription factors, and histone modification enzymes on the lung adenocarcinoma and brain metastasis in the paired samples.

Primary antibody	**Catalog Number**	**Dilution Ratio**	**Manufacturer**
Epithelial markers			
E-cadherin monoclonal antibody	ab1416	1:100	Abcam plc., Cambridge, UK
Occludins monoclonal antibody	ab216327	1:200	Abcam plc., Cambridge, UK
Desmoplakin monoclonal antibody	ab16434	1:100	Abcam plc., Cambridge, UK
α-catenin polyclonal antibody	PA5-36,046	1:100	Invitrogen Co., CA, USA
β-catenin monoclonal antibody	ab32572	1:500	Abcam plc., Cambridge, UK
type IV collagen polyclonal antibody	ab6586	1:500	Abcam plc., Cambridge, UK
Mesenchymal markers			
N-cadherin polyclonal antibody	ab76057	1:100	Abcam plc., Cambridge, UK
Vimentin monoclonal antibody	ab92547	1:200	Abcam plc., Cambridge, UK
Fibronectin polyclonal antibody	ab2413	1:50	Abcam plc., Cambridge, UK
$\alpha5\beta1$ integrin polyclonal antibody	MAB1969	1:50	Chemicon Inc., CA, USA
$\alpha\gamma\beta6$ integrin polyclonal antibody	orb100289	1:500	Biorbyt LLC., MO, USA
type I collagen polyclonal antibody	ab34710	1:200	Abcam plc., Cambridge, UK
Transcription factors			
Snail polyclonal antibody	orb180479	1:500	Biorbyt LLC., MO, USA
Slug polyclonal antibody	GTX121924	1:100	GeneTex Inc., CA, USA
Twist polyclonal antibody	ab50581	1:500	Abcam plc., Cambridge, UK
ZEB1 monoclonal antibody	ab180905	1:500	Abcam plc., Cambridge, UK
FOXC2 polyclonal antibody	PA5-24,588	1:50	Invitrogen Co., CA, USA
Histone methyltransferase			
MLL4 monoclonal antibody	ab56770	1:200	Abcam plc., Cambridge, UK
RIZ polyclonal antibody	LS-C324276	1:100	LSBio Inc., WA, USA
EZH2 polyclonal antibody	ab191080	1:250	Abcam plc., Cambridge, UK
NSD2 polyclonal antibody	ab137429	1:100	Abcam plc., Cambridge, UK
Histone demethylase			
LSD1 polyclonal antibody	ab17721	1:50	Abcam plc., Cambridge, UK
JMJD1 monoclonal antibody	ab91252	1:500	Abcam plc., Cambridge, UK
UTX polyclonal antibody	ab235989	1:200	Abcam plc., Cambridge, UK
JMJD5 monoclonal antibody	sc-377,078	1:50	Santa Cruz Biotechnology Inc. TX, USA

Abbreviations: EMT, epithelial-to-mesenchymal transition; MET, mesenchymal-to-epithelial transition.

Table S2. The lists of primers for quantitative reverse transcription–polymerase chain reaction for mRNA of the candidate genes of transcription factors associated with epithelial-to-mesenchymal transition, and histone modification enzymes on the lung adenocarcinoma and brain metastasis.

Primer	Forward Sequence	Reverse Sequence
	Transcription factors	
Snail	TGCCCTCAAGATGCACATCCGA	GGGACAGGAGAAGGGCTTCTC
Slug	ATCTGCGGCAAGGCGTTTTCCA	GAGCCCTCAGATTTGACCTGTC
Twist	GCCAGGTACATCGACTTCCTCT	TCCATCCTCCAGACCGAGAAGG
ZEB1	GGCATACACCTACTCAACTACGG	TGGGCGGTGTAGAATCAGAGTC
FOXC2	TCACCTTGAACGGCATCTACCAG	TGACGAAGCACTCGTTGAGCGA
	Histone methyltransferase	
MLL4	GGAATGGGTAGCTCTTTGGCGA	TGCCGAATCAGCAGCTCTCGTA
RIZ	TTGGGCTTGCTCAGGAGAAGAG	GCTGCTATCTCAGGGTTGTCTTC
EZH2	GACCTCTGTCTTACTTGTGGAGC	CGTCAGATGGTGCCAGCAATAG
NSD2	TGTGTGAGCTGCCATGCTTCCA	TGAGCATCCTGCTGCCAGACAA
	Histone demethylase	
LSD1	TCAGGAGTTGGAAGCGAATCCC	GTTGAGAGAGGTGTGGCATTAGC
JMJD1	GCCAACATTGGAGACCACTTCTG	CTCGAACACCTTTGACAGCTCG
UTX	AGCGCAAAGGAGCCGTGGAAAA	GTCGTTCACCATTAGGACCTGC
JMJD5	CACAGATGAGGAATGGTCCCAG	GCTGATGTCCTGCTTCAACTCC
	Control	
GAPDH	GTCTCCTCTGACTTCAACAGCG	ACCACCCTGTTGCTGTAGCCAA

Publisher's Note: MDPI stays neutral with regard to jurisdictional claims in published maps and institutional affiliations.

© 2020 by the authors. Licensee MDPI, Basel, Switzerland. This article is an open access article distributed under the terms and conditions of the Creative Commons Attribution (CC BY) license (http://creativecommons.org/licenses/by/4.0/).
